# Advances in Kriging-Based Autonomous X-Ray Scattering Experiments

**DOI:** 10.1038/s41598-020-57887-x

**Published:** 2020-01-28

**Authors:** Marcus M. Noack, Gregory S. Doerk, Ruipeng Li, Masafumi Fukuto, Kevin G. Yager

**Affiliations:** 1 The Center for Advanced Mathematics for Energy Research Applications (CAMERA), Lawrence Berkeley National Laboratory, Berkeley, CA 94720 USA; 2 Center for Functional Nanomaterials, Brookhaven National Laboratory, Upton, NY 11973 USA; 3 National Synchrotron Light Source II, Brookhaven National Laboratory, Upton, NY 11973 USA

**Keywords:** Energy science and technology, Materials science, Mathematics and computing

## Abstract

Autonomous experimentation is an emerging paradigm for scientific discovery, wherein measurement instruments are augmented with decision-making algorithms, allowing them to autonomously explore parameter spaces of interest. We have recently demonstrated a generalized approach to autonomous experimental control, based on generating a surrogate model to interpolate experimental data, and a corresponding uncertainty model, which are computed using a Gaussian process regression known as ordinary Kriging (OK). We demonstrated the successful application of this method to exploring materials science problems using x-ray scattering measurements at a synchrotron beamline. Here, we report several improvements to this methodology that overcome limitations of traditional Kriging methods. The variogram underlying OK is global and thus insensitive to local data variation. We augment the Kriging variance with model-based measures, for instance providing local sensitivity by including the gradient of the surrogate model. As with most statistical regression methods, OK minimizes the number of measurements required to achieve a particular model quality. However, in practice this may not be the most stringent experimental constraint; e.g. the goal may instead be to minimize experiment duration or material usage. We define an adaptive cost function, allowing the autonomous method to balance information gain against measured experimental cost. We provide synthetic and experimental demonstrations, validating that this improved algorithm yields more efficient autonomous data collection.

## Introduction

A central goal in experimental material science is to explore and understand the composition-processing-structure-property relations of materials in their associated multi-dimensional parameter spaces^1–3^. These parameter spaces can be thought of as the set of all conceivable combinations of the parameters affecting an experiment, including synthesis and processing conditions, material composition, and environmental conditions during the experiment. In an attempt to characterize a material—that is, to explore the parameter space—scientists traditionally change the parameters of the experiment interactively; when one measurement is accomplished, the recent and all prior results are interpreted and used to manually assess trends in the data, which are then utilized to determine the next measurement parameters. This manual approach is not only costly in the sense that it consumes valuable equipment and researcher time, but is also entirely insufficient when attempting to explore the vast, high-dimensional parameter spaces that underlie complex materials.

A properly explored parameter space means, mathematically, that we can, with high confidence, define a function that maps the position in this space onto a set of real numbers representing the quantities that the experimental instrument measures. For instance, suppose one is interested in doing an experiment in which the synthesis of a material can be performed at a range of temperatures (*T*) and the measurements can be performed at different sample locations [*x*, *y*]^*T*^. In this case, the experiment probes a three-dimensional parameter space ${\mathbb{P}}$. If, for instance, the measured quantity of interest is the material’s degree of crystallinity, say *ρ*, the goal is to find the function $\rho ({\bf{x}},T):{\mathbb{P}}\subset {{\mathbb{R}}}^{3}\to {\mathbb{R}}$.

For low-dimensional parameter spaces, experimental scientists traditionally sample the space by selecting a grid of experimental conditions, with grid spacing selected somewhat arbitrarily. For 1 − 3 parameters, this method is manageable; for  > 3 parameters, the procedure becomes increasingly ineffective and impractical. In these cases, a common method of experimental guidance is to determine the next measurement using intuition based on past measurements and the experimenter’s knowledge/experience. For a small number of parameters and an experiment that has been performed extensively before, this approach can be highly successful; however, for new experiments it can introduce a strong bias and potentially fails to discover new science in unexpected parts of the parameter space. Also, this method is rather costly, because it needs the constant attention of a human expert to determine the next, often non-optimal, measurement. And last but not least, the intuition-based and grid-based approaches provide no quantitative measure to decide when the experiment can be terminated.

Rapidly advancing computing power and instrumentation efficiency makes it increasingly important to be able to perform experiments quickly and autonomously. This large-scale automation and optimization allows for more complex scientific challenges to be explored by minimizing the number of data points needed to fully characterize a system. These important experimental issues serve as the motivation for the work on methods for optimal and autonomous experimentation.

Design of experiment (DOE) methods seek to find optimal measurement schemes^4^. These methods are largely geometrical, referred to as static sampling methods since they are independent of the measurement outcome and are concerned with efficiently exploring the entire parameter space. The Latin hyper-cube technique is the prime example of this class of methods^5,6^. When the optimization of a specific feature of a material is the goal, a one-variable-at-a-time (OFAT/OVAT) approach^7^ is often employed. This method fails, however, for non-convex or non-concave model functions, i.e. function that exhibit second derivatives that change signs. Most of the recent approaches to steer experiments fall into the category of dynamic sampling algorithms and are largely based on machine learning techniques, in which data is used for the machine to learn about a model function^2,8–10^. The authors of Ref. ^8^, for instance, used a sparse supervised learning approach to find the most information-rich locations in order to minimize the dose in diffraction-based protein crystal positioning. The work in Ref. ^11^ utilized the power of a deep neural network to simulate costly measurements. Another, very efficient type of algorithms comes from the field of image reconstruction. Here, the goal is to minimize the number of measurements needed to recreate an image^9^. However, these methods are generally optimized to explore low-dimensional spaces. A useful collection of methods can be found in Refs. ^12^ and^13^.

**Figure 1:**
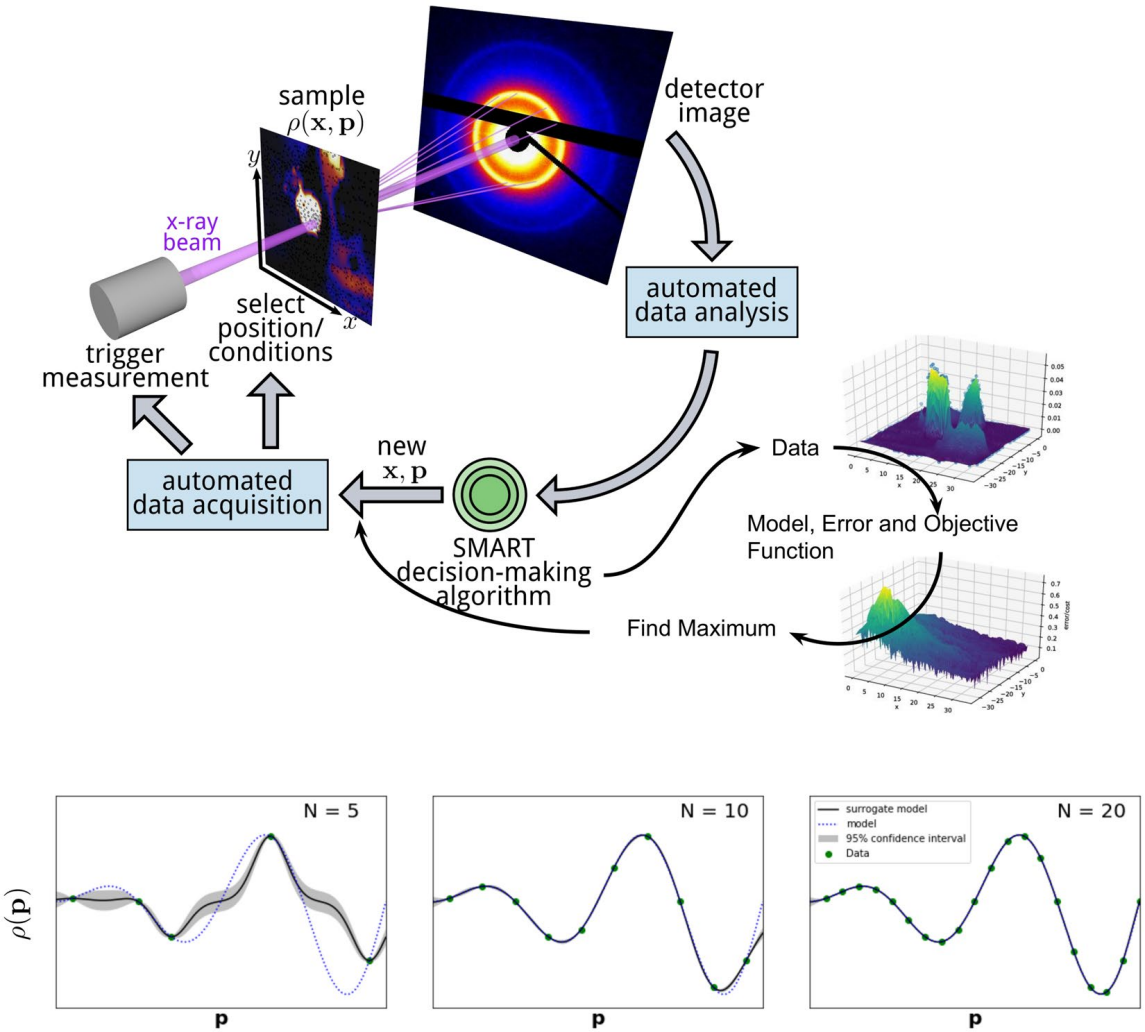
Schematic of an autonomous x-ray scattering experiment. When the measurement is performed, the data acquisition and data processing occur automatically. From the processed data, the SMART algorithm selects the next measurement parameters by finding an error function, a corresponding objective function and its maximum. Figure 2 provides a more detailed look into the way the data is used to find the optimal next measurement position. The model is computed as a byproduct in each step. The graphs (bottom) show an example where a one-dimensional problem is studied. As the number of measurements (*N*) increases, the surrogate model more and more closely matches the actual physical system behavior and the error (gray shade) decreases. For low data density, the actual model does not have to be inside the gray error bounds (far left). This is a common behavior which will not affect the autonomous experiment and it will disappear when more data is gathered.

Noack *et al*.^1^ explored a general approach to autonomous experimentation based on ordinary Kriging (OK), called SMART, which stands for Surrogate Model Autonomous expeRimenT. OK is able to efficiently generate a surrogate model function based on collected data, and compute a corresponding variance, which can be thought of as an error function that quantifies the uncertainty of the surrogate model. The next measurement should then be performed where the variance is estimated to be a maximum^14^, since this will decrease uncertainty the most and thus maximize information gain. This procedure is applied iteratively. After each measurement conducted at an uncertainty maximum, a new model function and variance are constructed. This way, the autonomous procedure iteratively decreases the error of the surrogate model. Figure 1 shows a schematic of the autonomous experiment procedure. It was shown that the method is able to efficiently uncover the correlations between measurable properties and a set of experimentally controllable parameters (temperature, pressure, etc.) by means of a surrogate model. Using the data of all past measurements, the method rapidly reduced the initial error of the model and was able to find a high-confidence model quickly. Additionally, the method returns an estimated error after each iteration, providing a convenient termination criterion.

However, comprehensive testing has also uncovered several limitations of the SMART method. OK is able to perform regression very efficiently by utilizing a stationary kernel, called the variogram. The variogram is found by fitting a predefined parametric function to the relationship of data differences and distances. Without user input, the function is fitted to the global data set and can therefore not contain any information about local data variation. Therefore, OK exclusively takes into account global variations of the data. In statistics, this phenomenon is known as stationarity; the mean is an unknown constant within the domain, and the difference in the data only depends on the distance, not on the respective location of the data within the domain. OK, without additional experiment-specific tuning, therefore assumes first and second order stationarity of the data, which cannot be guaranteed in an autonomous experiment. The stationarity requirements mean that OK cannot take into account local features of the model, such as local high gradients.

Even though many improvements have been proposed for Kriging (e.g. universal Kriging), they are either not accomplished autonomously or use the entire armada of Gaussian process regression methods which are computationally more costly. For instance, whenever autonomy is not required, the user can predefine a length-scale to make the variogram more sensitive to local data, which is not an option for autonomous experiments.

**Figure 2:**
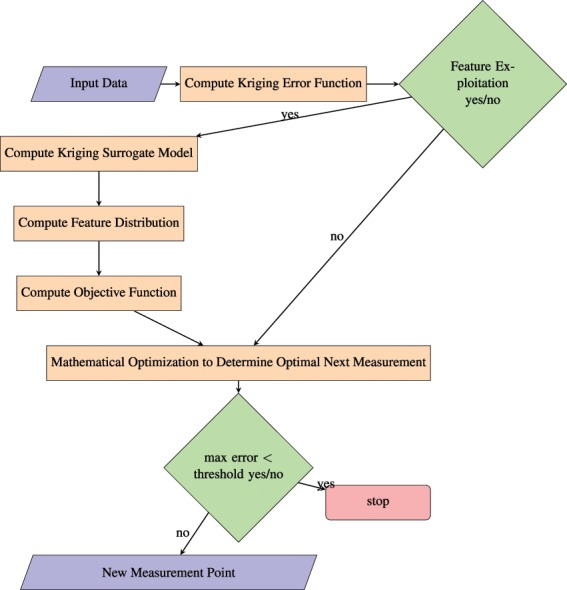
A flow chart depicting, in detail, the path from data to the next optimal measurement point. This figure should be interpreted as an extension to Fig. 1. Note that this paper is concerned with the left column of the flow chart. The Theory section will guide the user through each step in the flow chart.

The purpose of this work is to augment the OK error function, to create an objective function that reflects local information of the model. This local information will be referred to as "features" throughout the paper, and the particular examples of gradients and function values will be investigated. The OK error function, together with a measure of these features will be used to define an objective function, whose maximum constitutes an optimal next measurement location. Refer to Figure 2 for a schematic of this process. Additionally, instead of selecting measurements by finding the maximum of an error function that corresponds to the model, we explore an alternative approach of finding the maximum error per experimental cost. The error function is used to alter the objective function which results in optimal experiments with respect to a defined cost measure.

The proposed method is closely related to Bayesian optimization^15–17^. In particular, making use of the function values for the computed surrogate model is a well-understood approach in Bayesian optimization. While Bayesian optimization mainly works with the function value of the model, in this paper, the main goal is to provide the experimentalist with a general and intuitive framework to make a Kriging-based autonomous search algorithm more sensitive to a variety of features of interest and to handle measurement costs. Also, the proposed method provides a convenient way to shift between exploration and exploitation of the autonomous experiment, without any user interaction.

This paper is organized as follows. First, the derivation of the necessary theory of ordinary Kriging will be repeated for convenience, adding the treatment of local features of the model and the costs. Next, several synthetic tests based on a purposefully chosen test function will be presented to showcase the functionality of the new features. The last section shows the results of an x-ray scattering experiment at a synchrotron beamline, which took advantage of some of the proposed methodology.

## Theory

We first present a concise derivation of the ordinary Kriging variance (or just Kriging variance) *σ*^2^(**p**), which we will refer to as the “error function” throughout this paper. We will then augment the error function, using local information and word measurement costs, to create an improved objective function, whose maximum constitutes the optimal selection for the next measurement. Local features (e.g. gradients) are included by introducing a probability density function that defines a probability of finding certain values of the feature within the domain. This probability is then used to make decisions regarding the use of the feature for steering. Costs are included by defining local cost functions, whose global minimum is located at the last measurement point. In other words, local cost functions define the cost of movement in ${\mathbb{P}}$ away from the position of the most recent measurement. The offset of the local cost function defined the average cost of a measurement.

### Derivation of the Kriging variance

Ordinary Kriging, an instance of Gaussian process regression, is used to compute an interpolant that inherently minimizes the estimated variance between the data points. Kriging constructs the surrogate model function as a linear combination of weights *w*(**p**) and data points *ρ*(**p**_*i*_), where *i* is the index of the *i*th measured data point, not the *i*th component of **p**. In an imaging context, the location in the image **x** is contained in **p**. The surrogate model function is defined by

1 $${\rho }_{s}({\bf{p}})=\mathop{\sum }\limits_{i}^{N}{w}_{i}({\bf{p}})\ \rho ({{\bf{p}}}_{i}),$$

where *ρ*(**p**_*i*_) are the measured values of the true physical model *ρ* at point **p**_*i*_, obtained from previous measurements. Kriging is based on minimizing the mean squared prediction error (see Ref. ^18^ for details)

2 $${\sigma }^{2}({\bf{p}})={C}_{00}-{{\bf{w}}}^{T}{\bf{C}}{\bf{w}}-2{{\bf{w}}}^{T}{\bf{D}},$$

where the matrix **C** and the vector **D** are defined as

3 $${C}_{ij}=1-\gamma (| | {{\bf{p}}}_{i}-{{\bf{p}}}_{j}| {| }_{{}_{2}}),$$

4 $${D}_{i}=1-\gamma (| | {\bf{p}}-{{\bf{p}}}_{i}| {| }_{{}_{2}}),$$

where again, **p** refers to the position in ${\mathbb{P}}$ where the error is to be estimated, and *γ* is the so-called variogram. The matrix **C** is the covariance matrix that contains the correlations between all points in the data set. The vector **D** contains all correlations between the points in the data set and the point to be estimated. Since **C**^−1^ is required in the calculation, the method has numerical complexity *O*(*N*^3^), where *N* is the number of measurements.

**Figure 3:**
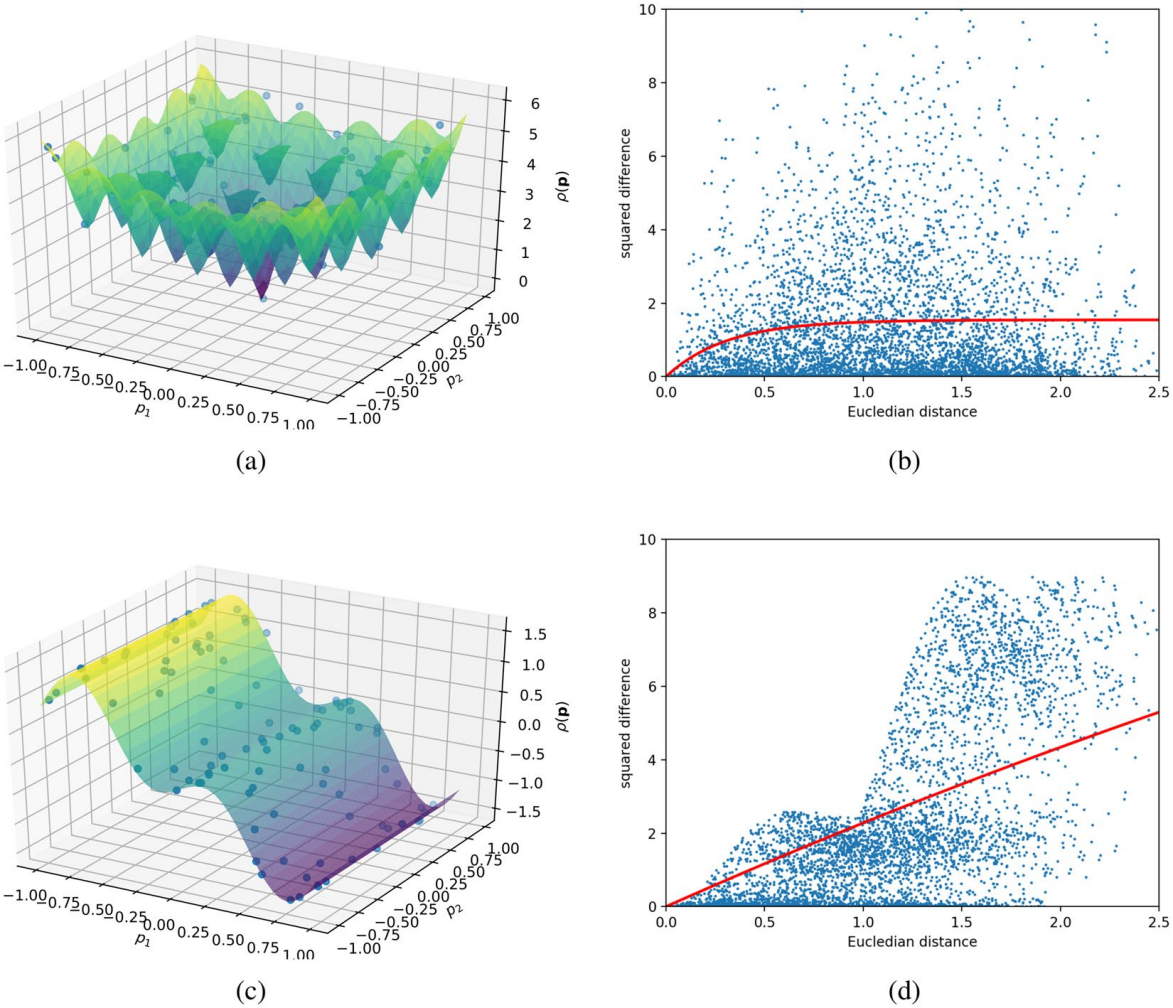
Variograms for two test functions and random measurement points (blue dots in (**a**) and (**c**)). Variograms serve as a measure for the dependency of data correlation on distance and play a vital role in Kriging. The dots in (**b**,**d**) show the difference of the data, defined by $\Delta {\rho }_{ij}={(\rho ({{\bf{p}}}_{i})-\rho ({{\bf{p}}}_{j}))}^{2}$. The red lines represent the fitted variograms. The shape of the variogram is found by a least-squares fitting of a parametric function to the squared difference of the data points. This fitting is repeated periodically to account for the most recent data. Note that **C** and **D** in Eqs. 3 and 4 are functions of the variogram. A mostly-flat variogram, that reaches a seemingly-constant value at low distances, translates into a strong correlation of data points. A steeply increasing variogram value over all distances translates into statistical low correlation of data points. (**a**) Test function portraying a mathematical model with correlations at many different length scales. This function is the well-known Ackley’s function. (**b**) Variogram, *γ*, for Ackley’s function in (**a**). Model function values are strongly correlated locally, and also over large distances. The average squared distance (red line) suggests that local information can be extrapolated to remote parts of the parameter space. (**c**) Test function portraying a mathematical model with small correlations over large distances. (**d**) Variogram, *γ*, for the synthetic test function in (**c**). While some model function values are highly correlated, there is a large set of pairwise comparisons where the correlation is very poor. The average behavior (red line) suggests that one needs to collect information over relatively short distances in order to confidently reconstruct the model.

In this work, the variogram is defined as

5 $$\gamma (h)=1\ -\ {e}^{-lh},$$

where *h* is the Euclidean distance between two points $||{{\bf{p}}}_{1}-{{\bf{p}}}_{2}|{|}_{{}_{2}}$. The variogram in Eq. 5 is referred to as exponential kernel in the Gaussian process literature. Other variograms can be considered. See Ref. ^19^ for a comprehensive overview of kernels. The variable *l* is chosen in a least-squares manner to fit the squared difference of the data (see Figure 3). Local constraint minimization of Eq. 2 via the Lagrange multiplier technique^18^, yields the equation for the weights

6 $${\bf{w}}={{\bf{C}}}^{-1}({\bf{D}}-\lambda {\bf{1}}),$$

where

7 $$\lambda =\frac{{{\bf{D}}}^{T}{{\bf{C}}}^{-1}{\bf{1}}-1}{{{\bf{1}}}^{T}{{\bf{C}}}^{-1}{\bf{1}}}.$$

**1** in Eqs. 6 and 7 is a vector of 1s. The estimator has to be unbiased, therefore we assume ∑*w*_*i*_ = 1, which serves as the constraint in the optimization. A major take-away from Eq. 6 is that the weights can be entirely determined by the geometry of the data and the point at which we want to estimate the surrogate model function. Inserting Eqs. 6 and 7 in Eq. 2 yields the final expression for the error or the so-called ordinary Kriging variance

8 $${\sigma }^{2}({\bf{p}})={C}_{00}-{\bf{w}}{({\bf{p}})}^{T}{\bf{D}}({\bf{p}})-\lambda ({\bf{p}}),$$

which we will refer to as (Kriging) error function throughout this paper. The main goal, in this work, is to augment the Kriging error function in Eq. 8 to account for local features of the model and the costs of the measurements.

### Accounting for features of the surrogate model

Here we introduce a simple method to incorporate local information about the surrogate model function for steering autonomous experiments efficiently. This is done by augmenting the original error function in Eq. 8 by terms encoding the desired feature. The challenge is to make decisions about how strongly to emphasize the selected feature. We accomplish this by making use of probability density functions for the feature. For illustrative purposes, we will deal with the specific example of the absolute value of the gradient and the function value of the surrogate model function as the “features” of interest where applicable. The absolute value of the gradient is useful when the experimental aim is to find regions of rapidly changing characteristics (e.g., phase boundaries); while the function value can be used to home in on the specific set of parameter values that optimize a material property (e.g., largest grain size). Instead of using the absolute value of the gradient one can only use a single component of the gradient if desired. We will therefore, from here on out, not make a distinction between a component of the gradient or the absolute value, and just refer to it as gradient.

**Figure 4:**
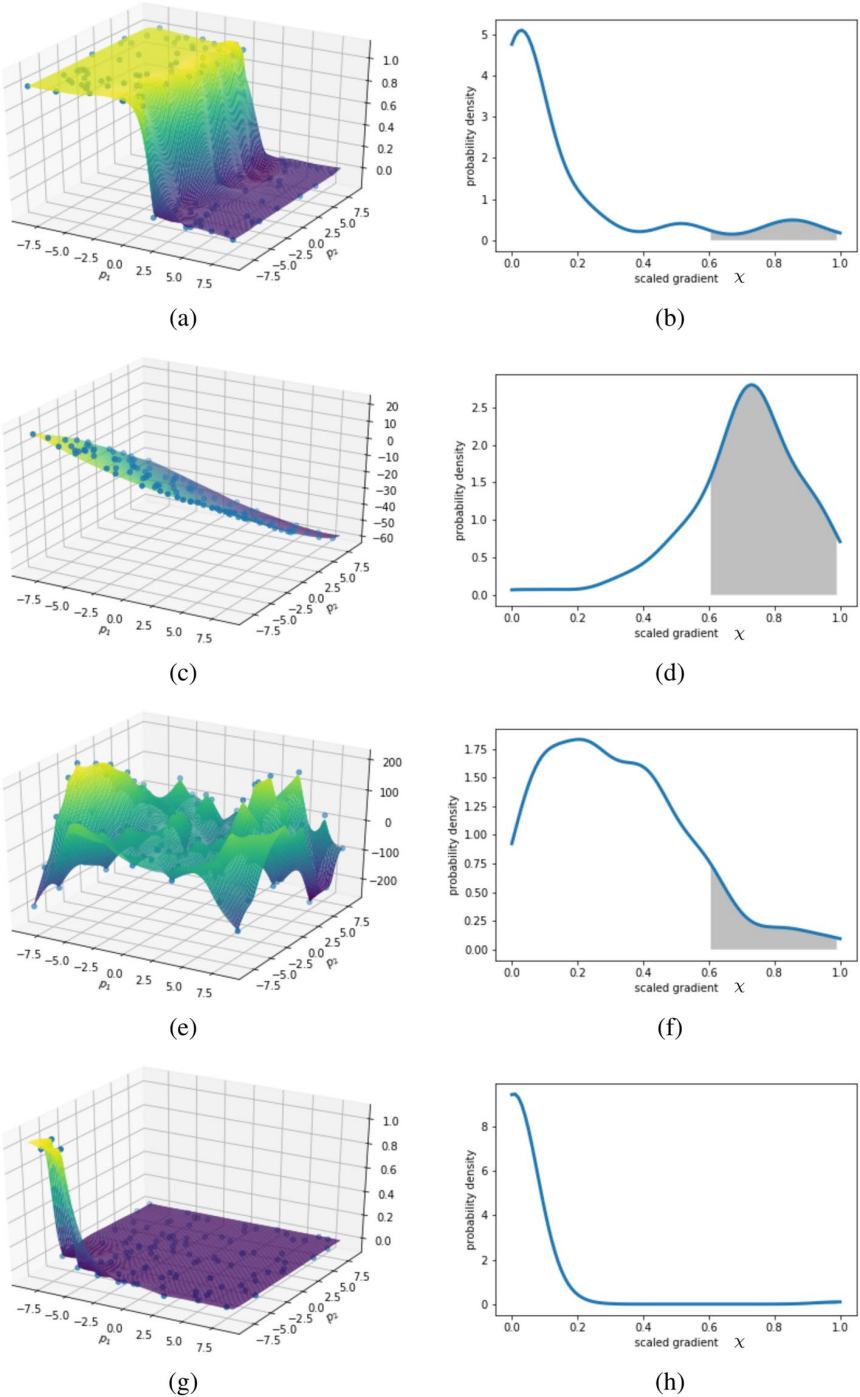
Four different test functions and the corresponding probability densities of the gradient. The step function (**a**) is mostly flat but comprises a small region with exceptionally high gradients (see (**b**)). This is a prime example for invoking the gradient. The test function in (**c**) comprises almost entirely (comparably) high gradients (see (**d**)). Here, the gradient would not be used for steering. The third test function (**e**) comprises non-zero gradients almost everywhere and seemingly no extremely steep regions either, which is reflected by the corresponding distribution of gradients (**f**). In this case, it is up to the choice of the user-defined gray interval if the gradient is invoked. The last function (**g**) is an often-encountered problem in which the human expert wants to find small areas of interesting activity. Here, the gradient would be invoked, if the user defines small bounds on the integral in Eq. 9 (see (**h**)). The gray shaded area dictates how strongly the gradient is invoked; when this area is large the gradient should be ignored. The limits of the integral and its impact on the gradient can be defined by the user. The reader is encouraged to judge how strongly they would have invoked the gradient in each of the examples.

A feature evaluated at a large number of randomly chosen points throughout the parameter space (random according to a uniform probability density function), constitutes a random variable which defines a probability density function. This probability density function can be used to calculate the probability of finding the feature within selected limits. For instance, evaluating the absolute value of the gradient of the surrogate model at 1000 points will give a distribution, which can be used to calculate the probability that a newly chosen point shows a gradient within a given range. Figure 4 shows the probability density function (PDF) for randomly chosen gradients, where the gradient values for each test function are scaled to [0, 1], and each PDF integrates to unity (as required for probability density functions). From this PDF, our algorithm can make decisions on whether the chosen feature, here the gradient, should be taken advantage of or not. If the vast majority of gradients are relatively high, the algorithm should not focus on them since high-gradient regions are common. If, on the other hand, the vast majority of gradients are characterized as low and few gradients are characterized as high, the high gradients areas are exceptional and should be preferentially explored as features of interest. What constitutes “high” and “low” relative gradients can be defined by the user, not as absolute values but as relative values  ∈ [0, 1].

**Figure 5:**
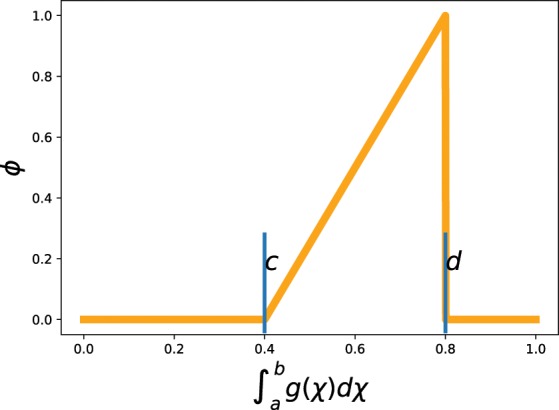
A graphical depiction of the function *ϕ* that is used to weight the impact of the feature on the final objective function. The function shows that only certain probability values (abscissa) will result in invoking the gradient. Within the limit [*c*, *d*], the dependence is linear. *c* and *d* can be defined by the user, as well as the boundaries of the integration domain *a* and *b*.

The described procedure is used for both the gradient and the function value of the surrogate model function. A measure of how much emphasis should be placed on a given model feature in steering the experiment can then be defined by the following unitless functional:

9 $$\phi (g(\chi );a,b,c,d)=\left\{\begin{array}{l}0\ for\ {\int }_{a}^{b}\ g(\chi )\ d\chi \  < \ c\\ 0\ for\ {\int }_{a}^{b}\ g(\chi )\ d\chi \  > \ d\\ \frac{{\int }_{a}^{b}\ g(\chi )\ d\chi \ -\ c}{d-c}\ else\end{array}\right.$$

where the function *g*(*χ*) is the PDF for the gradient or function value of the model, as a function of the scaled gradient *χ* (see Fig. 4), and *a*, *b*, *c* and *d* are user defined constants  ∈ [0, 1]. Naturally, *a* and *b* must have the unit of *χ* and *c* and *d* are unitless probabilities. It is important to note here that the choice of the constants does not depend on the unknown final model function, which would be undesirable, but only on the overall goal of the experiment. In other words, knowledge of the outcome of the experiment is not strictly required, but can be used if it exists. Intuitively speaking, the constants *a* and *b* are a way to communicate for the user which relative feature range constitutes high or low values. If, for instance, the user is looking only for areas where the gradient is significantly higher than the average, these values should be close to 1 (*a* < *b*). The values *c* and *d* are a way to express how common these features are. For example, if the user is interested in certain gradients which are very sparse, then possible values would be *c* = 0.2 and *d* = 0.6. In this case, when the feature is too common, it will be ignored. As for the sensitivity of the steering with respect to those parameters, it can be said that reasonable values will lead to an improvement of the resolution of the model. In the worst case scenario, the ranges for the features, specified by the users are too dense or sparse, such that the algorithm converts word to pure ordinary Kriging. *ϕ* will later serve as a coefficient to weight the impact of the feature on the final objective function, whose maximum will dictate the location of the next measurement (see Fig. 5). Eq. 9 is a piecewise linear function of the probability for a certain range [*a*, *b*] of the feature.

### Accounting for the costs of measurements

**Figure 6:**
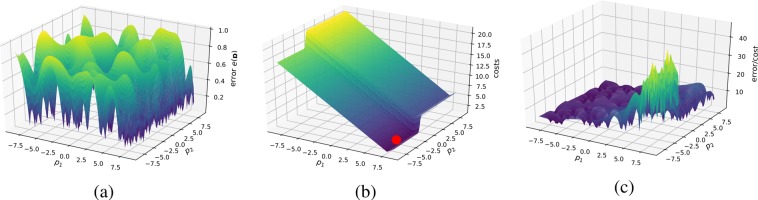
Typical error and cost functions and the resulting objective function. Note that the cost function depends on the location of the last measurement in the parameter space and changes with each new measurement. Therefore, finding the maximum of the objective function amounts to finding the best ratio of model accuracy improvement and cost efficiency. The position of the maximum of this function constitutes the optimal next measurement, which can be communicated to the measuring device. (**a**) The original error function *e*(**p**). (**b**) A typical local cost function. This cost function combines linear costs in the *p*_1_ direction, such as the one associated with sample movement, and a sigmoidal cost in *p*_2_ direction, associated with changing samples, for instance. It is centered around the last measurement points (marked). (**c**) The resulting objective function defined in Eq. 11. The objective function has the units of $\frac{error}{costs}$, where the error has the unit of the model itself.

Up to this point we have developed a method that will inherently minimize the estimated model error for a given number of performed measurements. However, in many cases in experimental sciences, the objective, in fact, is not to minimize the total number of measurements but some other quantity reflecting experimental costs, for instance, time or the use of a costly material. To address these cases, we introduce local cost functions which can augment the original error function. Costs are accounted for in the sense of local costs of moving to a new point **p** from the previous point $\widetilde{{\bf{p}}}$ in the experimental parameter space. For this we define a local cost function as

10 $$c({\bf{p}},\widetilde{{\bf{p}}})=c(| | {\bf{p}}-\widetilde{{\bf{p}}}| | )=\mathop{\sum }\limits_{i}^{N}\ {f}_{i}({p}_{i}-{\widetilde{p}}_{i}),$$

where ${f}_{i}({p}_{i}-{\widetilde{p}}_{i})$ are linear, sigmoid or other functions defining the cost of moving in the direction *p*_*i*_. Note here, that *p*_*i*_ now refers to the *i*th component of **p**. $\widetilde{{\bf{p}}}$ is the location of the last measurement point, while **p** is the new point at which we are computing the cost and later the objective function value. Therefore, the cost function is centered at the last measurement point and reflects the cost of moving to the next measurement location. In this case ∣∣ ⋅ ∣∣ is the *L*^1^ norm (for more information see Ref. ^20^), which is the most applicable for many experiments. This is due to the fact that the parameters of an experiment can often only be changed one at a time. For instance, if the parameters are the position of the motion stage at a beamline (e.g., for a different sample position), movement may need to occur in sequence (e.g. vertically then horizontally) rather than simultaneously (e.g. diagonally). The cost of moving a motion stage is approximately described by a linear function of the travel distance, while switching samples may be defined by a sigmoid function if the cost is independent of which new sample will be measured next (Fig. 6(b)). One could also use a step function in this case, but maintaining differentiability for the cost function can be advantageous as it will keep the final augmented objective function, to be described next, differentiable. Maintaining differentiability is necessary if local optimization algorithms, like gradient based optimization methods are used. Note that when using linear cost functions, differentiability is not provided and derivative-free optimization techniques have to be used.

### Combining variance, features and costs into one objective function

When combining the Kriging error function, the features of the surrogate model and the costs into one equation, we have to ensure that their associated units make physical sense. The Kriging variance, or error function, has the unit of the model squared ([model^2^], for example nm^2^ for grain size), the surrogate model function has the unit of the model (in this example [nm]), the gradient has the model unit per distance in parameter space ${\mathbb{P}}$ (e.g. [nm ∕mm]) and the costs have assigned units (minutes, dollars, etc.). Since a measurement point $p\ \in \ {\mathbb{P}}$ should be selected to improve the surrogate model, we can intuitively state that we want to perform the next measurement where the $\frac{error\ (improvement)}{cost}$ is a maximum. Therefore, the objective function, which we want to maximize, can be defined as

11 $$F({\bf{p}},\widetilde{{\bf{p}}})\ =\ \frac{\sqrt{{\sigma }^{2}({\bf{p}})}+{\phi }_{1}| \nabla \rho ({\bf{p}})| \frac{n}{2}+{\phi }_{2}\rho ({\bf{p}})}{c(| | {\bf{p}}-\widetilde{{\bf{p}}}| | )},$$

where *n* is the nearest-neighbor distance averaged over all points in the data set (respective points with smallest Euclidean distance). Eq. 11 now has the desired unit of error in the model unit per cost. The *n* term balances units by selecting an appropriate scale by which we combine model values and gradients thereof; *ϕ*_1∕2_ are defined in Eq. 9. In this case, we are using two features with their respective distributions, e.g. the gradient and function value of the surrogate model.

The added features of interest—the gradient and the function value of the surrogate model are used here—raise the function value of the objective function; therefore, regions where the feature is present are preferred as the next measurement location. The objective function will have lowered function values in regions where the cost is high, leading to a preference for the next measurement where the cost is low, thereby maximizing the information gain per cost (see Fig. 6) per measurement.

### Maximizing the objective function

When optimizing the highly non-linear objective function, we have to strike an optimal trade-off between computational efficiency and functionality. Noack *et al*.^1^ employed a genetic algorithm to quickly find a suitable solution. The use of other global optimization methods, like differential evolution, are acceptable. The genetic algorithm and differential evolution are ideally suited when the dimensionality of ${\mathbb{P}}$ is low and only one, potentially local, maximum is sufficient. While the global optimum is the location of the optimal next measurement, any local optimum is an admissible solution. In low dimensional spaces, these algorithms can deliver a maximum very efficiently, which is preferred when many measurements have to be performed in a short amount of time. If many local maxima are sought, a purely global optimization method cannot guarantee to deliver. In this particular case, the HGDN algorithm^21^ is a good choice since it can find and eliminate optima, by using deflation. After deflation, the optimum cannot be found again by a Newton-based optimization. The optima (here maxima) of the objective function can then be provided to the measurement instrument. After each measurement, the updated data set is then used to create a new error and objective function.

## Synthetic Test

**Figure 7:**
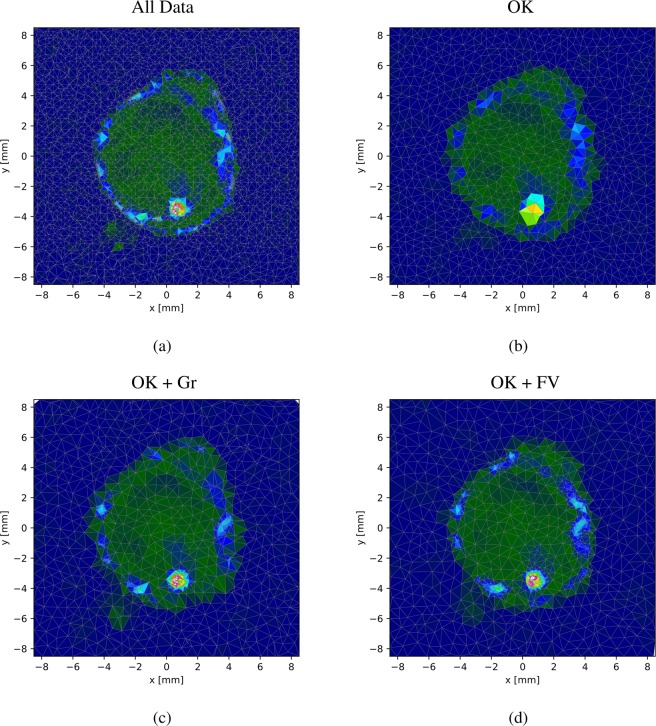
The test function used for all synthetic tests and its approximation using different versions of the proposed method. The test function was created by using all 4025 data points. The approximations use 1000 data points. Note, that even though the data amount is reduced to about 25%, the gradient and function-value supported results show a higher resolution in some of the areas of interest. (**a**) The test function created by using all 4025 available data points. (**b**) The approximation of the test function using ordinary Kriging. (**c**) The approximation of the test function using ordinary Kriging supported by gradient information. (**d**) The approximation of the test function using ordinary Kriging supported by function value information.

To highlight the different features of our proposed advancements of ordinary Kriging for autonomous experiments, we present a side-by-side comparison for each proposed improvement based on synthetic tests. We will refer to ordinary Kriging often simply as Kriging or abbreviate it by OK. The test function used is shown in Fig. 7, together with some illustrations showing the respective surrogate model for each of the method improvements. This test function arose from an actual x-ray scattering experiment performed at the CMS beamline at the National Synchrotron Light Source II, Brookhaven National Laboratory^1^. The sample comprises large regions of approximately zero function value and gradients, and limited regions of high-gradient and high-function-value regions. It is therefore ideally suited for our synthetic tests. All figures shown are the result of applying the proposed methods to explore the same test function, based on linear interpolation of the measured data points.

### Kriging vs gradient-supported and function-value-supported Kriging

**Figure 8:**
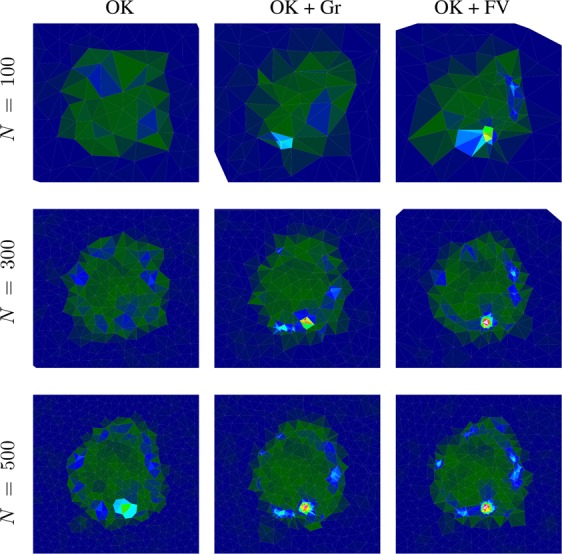
Measurement distribution and surrogate model function after 100, 300, and 500 measurements using ordinary Kriging (OK), ordinary Kriging with gradient support (OK + Gr) and ordinary Kriging with model-function-value support (OK + FV). Note how the definition of certain features of the model function of gradient and function-value supported calculations surpass the quality of ordinary Kriging.

**Figure 9:**
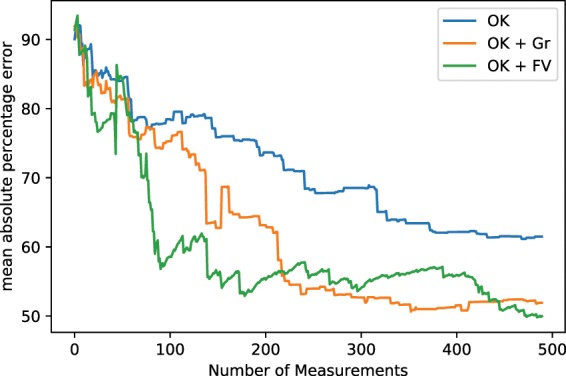
The mean absolute percentage errors corresponding to the experiments shown in Fig. 8. Note the increased performance of gradient and function-value-supported ordinary Kriging, which is in agreement with the message conveyed in Fig. 8.

First, we want to compare the results of pure Kriging, gradient-supported Kriging and function-value supported Kriging. The three algorithms were challenged to approximate the test function depicted in Fig. 7. The results are presented in Fig. 8. For this comparison the synthetic autonomous experiment was terminated after 500 measurements. The decrease in the corresponding mean absolute percentage errors with the number of measurements is shown in Fig. 9. Both figures clearly show that the quality of the gradient and function-value supported approaches outperform pure Kriging. This is due to the fact that, after an initial period, the supported algorithm can target specific regions which will contribute positively to the accuracy of the approximation. The values in Eq. 9 were chosen as follows: *a* = 0.8, *b* = 1.0, *c* = 0.02, *d* = 0.5.

### Kriging vs cost-constrained Kriging

**Figure 10:**
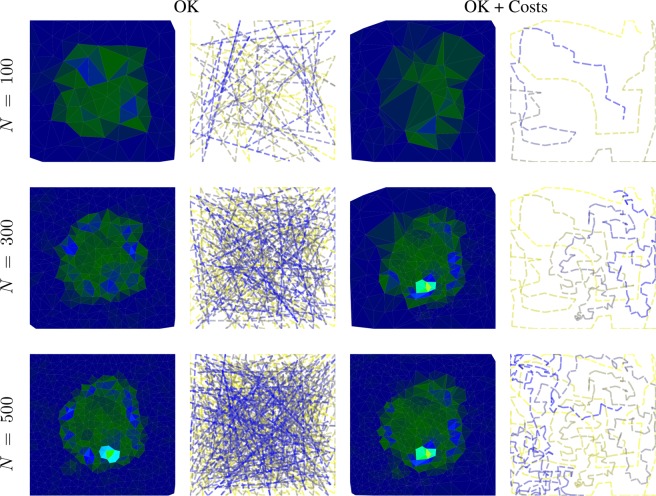
Model, measurement distributions and movement paths after 100, 300, and 500 measurements using ordinary Kriging (OK) and OK + Costs. Contrary to ordinary Kriging, cost-supported ordinary Kriging organizes subsequent measurements in a line to save movement costs. The color of the lines are for optical assistance to see the position of the measurements in order (blue to yellow and opaque to transparent).

**Figure 11:**
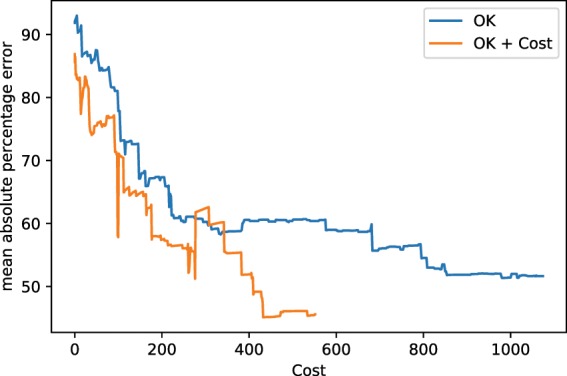
The mean absolute percentage errors corresponding to experiments shown in Fig. 10. Both experiments were run up to 500 measurements. Note that, especially in the beginning of the experiment, the cost-supported method reaches the same error at less cost.

This comparison is not concerned with the quality of the reconstruction, but rather with its efficiency. As described in the Theory section, the error function can be adjusted, so that the maximum in the objective function represents the best error improvement per cost. We again terminated the synthetic autonomous experiment after 500 measurements and the results are summarized in Fig. 10. The corresponding mean absolute percentage errors are displayed in Fig. 11. The two figures convey how choosing measurements by maximizing the error improvement per cost can make the autonomous experiment more efficient. Figure 10 shows that measurements are organized along a curve to minimize cost of movement. Figure 10 shows the result of this procedure; lower errors are reached at lower costs. The cost, in this example, was implemented as directional distance (*L*^1^).

### The role of costs in feature-supported Kriging

**Figure 12:**
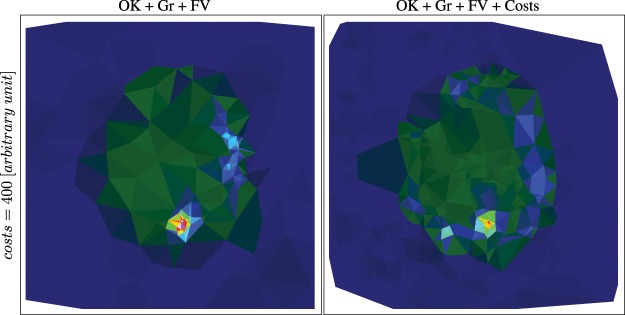
Model and measurement distributions after a cost of 400 using ordinary Kriging supported by gradients and function values (OK + Gr + FV) versus the same, constrained by costs (OK + Gr + FV + Costs). Note the higher average resolution in the cost-constrained case. However, the resolution in high-gradient and high-function-value regions is slightly lower.

This comparison is concerned with the combination of feature-supported (here function-value and gradient supported) and cost-constrained Kriging. We compare the models after a certain cost spent, not after a certain number of measurements. The results are summarized in Fig. 12. The figure shows that a high resolution can be reached more efficiently by using costs in combination with function value and gradient support. However, the result also shows that the high resolution is more spread out and not as focused. This is due to the cost constraint, which causes the possible optimal-next-measurement point to not move freely.

### Kriging vs cost-constrained Kriging in three dimensions

Noack *et al*.^1^ showed ordinary Kriging applied to a three-dimensional physical test function which is defined as the diffusion coefficient *D* = *D*(*r*, *T*, *C*_*m*_) for the Brownian motion of nanoparticles in a viscous liquid consisting of a binary mixture of water and glycerol:

12 $$D=\frac{{k}_{B}\ T}{6\pi \mu r},$$

where *r* ∈ [1, 100] nm is the nanoparticle radius, *k*_*B*_ is Bolzmann’s constant, *T* ∈ [0, 100]° C is the temperature and *μ* = *μ*(*T*, *C*_*m*_) is the viscosity (Ref. ^22^), where *C*_*m*_ ∈ [0.0, 100.0] % is the glycerol mass fraction.

**Figure 13:**
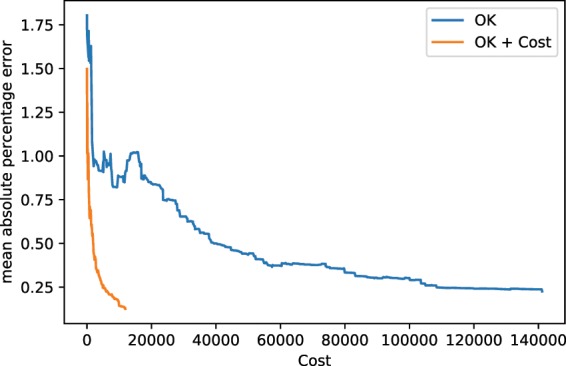
Mean absolute percentage error for a three-dimensional physics-inspired synthetic test function. The costs describes the total distance traveled. The cost-supported Kriging can lower the error much faster compared to Kriging without the cost support.

The diffusion coefficient of nanoparticles in complex fluids can be measured by x-ray photon correlation spectroscopy (XPCS), a coherent x-ray scattering method, which is available at modern x-ray light sources^23,24^. The dimensionality of this example emphasizes the need for autonomously steered experiments. The error convergence can be seen in Fig. 13. The error was defined as proportional to the directional distance (*L*^1^). The costs, however, significantly vary in different directions, as it is common for many experiments. This example shows how beneficial the inclusion of a cost function can be when the dimensionality of the parameter space increases.

## Experimental Validation

The presented methods were employed to conduct autonomous x-ray scattering experiment at the Complex Materials Scattering (CMS, 11-BM) beamline at the National Synchrotron Light Source II (NSLS-II), Brookhaven National Laboratory. Experimental control was coordinated by combining three distinct Python software processes: one controlling the beamline, one performing automated analysis of newly collected detector images, and one implementing the Kriging-based optimization presented herein. For the experiments discussed herein, transmission small-angle x-ray scattering (SAXS) data were collected using a two-dimensional area detector positioned 5.090 m downstream of the sample. The incident x-ray beam energy was 13.5 keV, and was focused to a spot size of 0.2 mm by 0.2 mm. The samples studied were self-assembling polymer thin films cast on a silicon substrate (0.2 mm thickness). In particular, the polymer films were block copolymers, which are self-assembling materials that spontaneously form well-defined nanostructures^25^. Films were applied using a novel positionally-controlled electrospray method^26^, allowing in-plane gradients in material composition to be created. This allows a single sample to represent a large library of different material compositions. The goal of the autonomous experiment was to measure gradient samples, in particular mapping the heterogeneity in ordering (as measured by x-ray scattering) both to probe the underlying materials physics, and to test and validate the deposition characteristics of the electrospray method. To illustrate this purpose, we present here data for a sample coated using non-optimal electrospray parameters, which thus displays both a smooth variation in material properties due to the composition gradient, as well as heterogeneity due to imperfections in the deposition.

The sample consisted of a ternary blend polymer film with gradient composition, deposited on a silicon substrate onto which a hydroxyl-terminated polystyrene-random-poly(methyl methacrylate) (PS-r-PMMA) copolymer brush with 61% PS content had been grafted^27^. The brush yields a chemically neutral substrate with respect to the ordering of the block copolymer film (PS-*b*-PMMA) that is subsequently deposited. The gradient polymer film deposition was accomplished using a custom-built combinatorial gradient electrospray deposition instrument, which is described elsewhere^26^. Briefly, immediately before spray deposition, polymer solutions were combined and mixed within a 50 mm long needle having a 100 *μ*m inner diameter orifice in proportions dictated by three automated, synchronized syringe pumps. Deposition was confined to a 1 mm diameter spot of electrosprayed material produced using a “small spot” extractor tube. An automated *x*-*y* stage translated the sample during spraying to deposit the polymers in a raster pattern of 32 mm long lines in the *y*-direction with 1 mm steps between them in the *x*-direction, thereby creating a 32 × 32 mm square pattern. Each spray line in the *y*-direction included a continuous gradient from 3.5 kg/mol PS homopolymer (*y* = 0 mm) to 3 kg/mol PMMA homopolymer (*y* = 32 mm); in the positive *x*-direction, a 104 kg/mol PS-*b*-PMMA lamellae-forming diblock copolymer was blended into the sprayed solution at increasing proportions from 0 to 100% that were constant within each spray line (3.125% steps per line). It is expected that composition steps in the x-direction are smoothed out by some small degree of overlap between adjacent spray lines. As a result, the target pattern is a square with pure block copolymer on one side (*x* = 32 mm) and pure homopolymer on the opposing side (*x* = 0 mm), where the homopolymer transitioned from PS to PMMA in the orthogonal *y*-direction. Overall, the sample thus represents a two-dimensional ternary phase diagram with every possible composition of the three mixed components (PS, PMMA, and PS-*b*-PMMA) represented.

All sprayed polymers were dissolved in propylene glycol monomethyl ether acetate (PGMEA) at a concentration of 1% (w/w) and solutions were sprayed at a rate of 10 *μ*L/min. For each gradient line, the substrate moved linearly at a speed of 0.15 mm/s. The substrate temperature was held at 150 ° C,  and extractor ring and nozzle voltages were 1 and 3.5 kV, respectively. After deposition, the PMMA polymer within the film was selectively infiltrated with aluminum oxide as described previously^28^ to increase X-ray scattering contrast.

**Figure 14:**
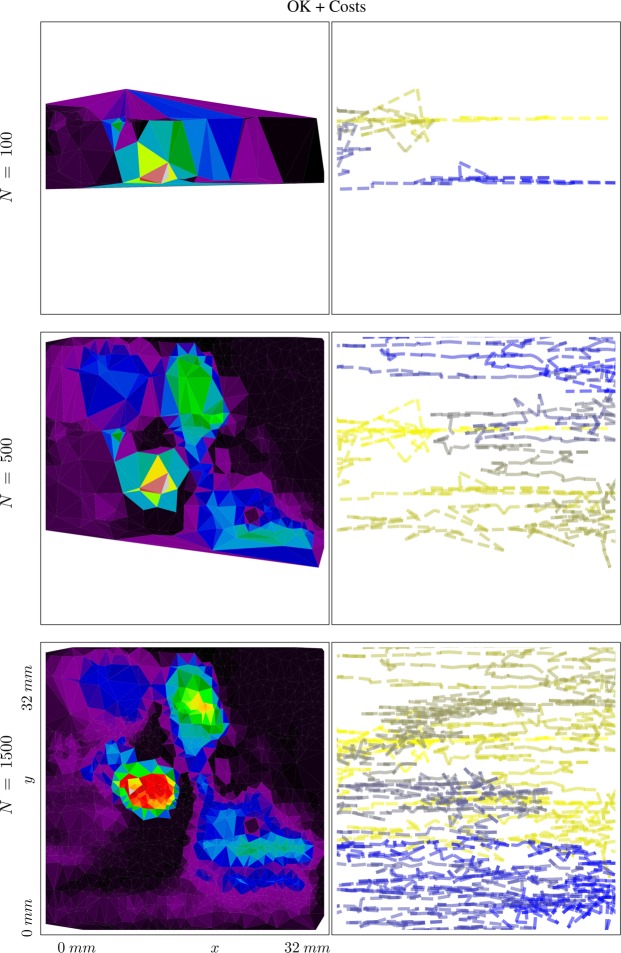
The resulting model for material ordering and the exploration path after 100, 500 and 1500 measurements (from top to bottom). The algorithm adjusted the cost function to account for the different speeds of the stage moving in different directions. Therefore movement in the horizontal direction in the figure is cheaper and therefore favored. The final cost function can be seen in Fig. 15. The color of the lines are of optical assistance to emphasise the path of the measurements.

**Figure 15:**
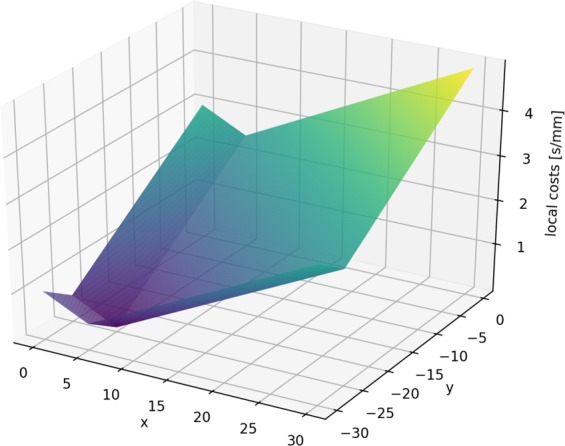
The cost function for the beam line experiment. Note that while a guess of the cost function has to be provided by the user, it will be updated as more data is recorded. The figure shows the final cost function centered at the final measurement point (i.e., the point of minimum cost). The algorithm reliably determined the different speeds of stage movement in *x* and *y* direction.

For this experimental exploration, the costs were of special interest. The experimental cost was calculated as the total time required to acquire a new datapoint, including motion of the sample to the new (*x*, *y*) coordinates selected by the algorithm, as well as the detector exposure time for the measurement. The algorithm presented here can be provided with an initial estimate for the cost function; however it will also track the returned experimental costs and compute an improved empirical cost model consistent with the actual measured costs. This cost update is done as follows: All measurement costs are recorded and outliers are removed. Periodically, a Newton optimization finds the parameters of the predefined cost function that best explain the recorded measurement costs. The influence of the cost modeling can be seen quite explicitly in Fig. 14. The cost keeps measurements “localized”, favoring new measurements that are close to the current (*x*, *y*) position. More interestingly, in this example the algorithm learned an anisotropic cost model; in particular determining that motion in the *x*-direction are lower-cost relative to motions in the *y*-direction. Correspondingly, the search path favored by the algorithm was to explore along a “stripe” at constant *y*, and move to a new *x* position only after sufficient exploration in this direction. This disparity in cost for the *x* and *y* directions was identified—after experiment conclusion—to be due to different motor speeds used to drive the two directions. Thus, the algorithm was able to learn and exploit a cost model that was not provided to it by the experimenters; indeed the difference was not known to the experimenters until after data collection was concluded. This emphasizes one key advantage of adaptive autonomous methods, in that they are able to both learn useful models for efficient constrained exploration of parameter spaces; and moreover that they do so adaptively and thus in a way that continually updates to match the experimental reality. The final local cost function is presented in Fig. 15.

The algorithm also efficiently reconstructs a model for material ordering. As can be seen (Fig. 14), the sample exhibited significant heterogeneity in the scattering intensity, with ordering being overwhelmed by regions of significantly higher scattering signal despite the underlying smooth gradient in material composition. This map suggested that the film was substantially thicker in localized regions, and was used to further optimize the electrospray deposition (and eliminate droplet formation which gave rise to these local defects). The autonomous algorithm was able to quickly identify heterogeneity, and localize these defects. Further measurements efficiently refined the delineation of these defects by adding new data points as necessary. Overall, this autonomous experiment demonstrates the utility of machine-guided exploration for providing experimenters with useful data, especially in cases where the experimenter cannot define ahead of time how the search should be performed. Moreover this test demonstrates the value of learned models for the surrogate, uncertainty, and cost; since adaptive models can react to unanticipated structure in the accumulated experimental data.

## Discussion

In this paper we have proposed several advancements to the ordinary Kriging method used to steer autonomous X-ray scattering experiments in Ref. ^1^. The first type of enhancements enables autonomous experimental modes that depend on local features of the surrogate model. The objective of such steering modes may be to optimize a certain measurable material property or to recognize and elucidate phase boundaries in the material parameter space. In these cases, the domain scientist knows that high function values or high gradient values in the surrogate model are the most important for steering the experiment, and this information can be used to make the autonomous experiment more efficient for a specific purpose of interest. Even if no prior knowledge about the model exists, the feature can be invoked if the user decides that the feature should be emphasized, contingent on its existence. Figures 8 and 9 show the improvement of the model quality given a number of measurements. For the examples studied here, the autonomous method converges to a low-error reconstructed model much more rapidly when exploiting these additional features. However, it is important to note that the success of the function-value and gradient-supported procedures depends highly on the character of the function being probed. If, during execution time, the chosen feature turns out not to be informative, the proposed algorithm will recognize it and cease to use the quantity. Therefore some model functions will not permit the feature to be used at all; the algorithm will, by itself, determine if invoking the selected feature is advantageous to the autonomous experiment outcome or efficiency. If not, the algorithm autonomously drops back to ordinary Kriging. Setting the user defined constants does not strictly require any prior knowledge about the model, but rather entails knowing which features of this model should be emphasized during steering. We want to emphasize that our treatment of local features can be included without adding significant computational costs. In cases where computational costs are not a limiting factor—such as when measurement suggestions are not needed rapidly—more sophisticated methods can be employed. Gaussian process regression is able to use non-local kernels which could, for instance, result in higher error function values in regions where the characteristic length scale is smaller. This commonly needs the maximization of a log likelihood, instead of a simple fitting of a variogram, which is a potentially expensive procedure. However, the authors are aware of these methods and will investigate and utilize them in future work.

The second type of advancement allows for the incorporation of experimental costs in guiding autonomous experiments. Figures 10 and 11 show how beneficial including cost can potentially be. Figure 10 shows how, under costs, measurements are autonomously organized in a pattern along a curve; thereby minimizing costly movement. Figure 11 shows the impact of the proposed method on the error of the autonomous experiment. The error drops more quickly compared to the same experiment without cost. Our treatment of costs is in the image of a greedy algorithm, i.e. the costs are re-evaluated in each iteration and measurement with the locally maximum improvement per cost is chosen. There is no guarantee that, looking back after a number of measurements, that, given the now known model, all measurements have been chosen to globally minimize the costs of the measurement. In other words, if we had known the model beforehand, we could have chosen more cost efficient measurements, a common issue with greedy algorithms. However, knowing the model beforehand would defeat the purpose of the experiment. In future work, one could include a global test function specified by the user that defines how the local cost functions change across the parameter space, depending on certain characteristics of experiments in different regions. For instance, in x-ray scattering, certain regions could require a longer exposure time than others, leading to higher measurement costs.

We want to emphasize that, even though we mostly have shown the new techniques being applied separately, the experimenter is free to combine features of the model and costs. Also, often it is desirable to switch between modes, which has shown to be very useful. This switch or a transition could be dependent on the number of measurements or on some preliminary interpretation of an early result. The take-home message here is that the proposed advancements of the Kriging-based autonomous experimentation can be combined or transitioned between dependent on the overall goal of the experiment.

One of the most challenging tasks we face when working on autonomous machines is that, contrary to many different fields, user interaction cannot be mandatory. Therefore, stability has to be the number one priority. One difficulty when invoking a feature of the surrogate model in the decision-making process is that it can lead to clustering, i.e., getting stuck in a certain confined region in the parameter space. When clustering becomes too strong, the correlation length of the data becomes very short and the prediction power of the algorithm actually decreases. Future work will therefore treat other ways of including local information. One possibility could be to use a non-stationary kernel which, unfortunately, as previously stated, is expected to come with high computational costs, which can however, partly be decoupled from the measurements.
